# Pharmacological Properties of *Platycarpha glomerata* Extracts—A Plant Used to Treat and Manage Elephantiasis

**DOI:** 10.3390/ijms26020646

**Published:** 2025-01-14

**Authors:** Siphamandla Q. N. Lamula, Aphelele Taliwe, Lisa V. Buwa-Komoreng

**Affiliations:** Infectious Diseases and Medicinal Plants Research Niche Area, Botany Department, Faculty of Science and Agriculture, University of Fort Hare, Private Bag X1314, Alice 5700, South Africa; 202014249@ufh.ac.za (A.T.); lbuwa@ufh.ac.za (L.V.B.-K.)

**Keywords:** anticancer, *Platycarpha glomerata*, antibacterial, antioxidant, FTIR, phytochemical analysis

## Abstract

*Platycarpha glomerata* (Thunb.) Less. has recently become a plant species of interest to researchers due to its biological activities and less toxic effects. Therefore, the aim of the study is to evaluate the in vitro anticancer potential and phytochemical constituents of *P. glomerata* plant extracts. Phytochemical screening and FTIR were carried out using standard methods. The antioxidant activity was accessed by determining its ability to scavenge the DPPH radical and nitric oxide radical, whereas the anticancer activity against prostate (DU-145 and PC-3), human T-lymphocyte (SKU-T), gastric adenocarcinoma (AGS), and human prostatic epithelial (PNTA1) cell line was evaluated using the MTT assay. The phytochemical analysis revealed the presence of tannins, flavonoids, saponins, steroids, terpenoids, and cardiac glycosides. The FTIR spectrum for the aqueous extract displayed characteristic peaks for O–H, C=O, C=C, and =C–H stretch. The aqueous ethanol and methanol extracts showed significant dose-dependent DPPH radical scavenging capacity. The aqueous, ethanol, and methanol extracts showed minimum NO scavenging activity of 4.3%, 9.6%, and 11.7% at 2500 µg/mL. The water extract demonstrated good activity against *S. aureus*, *E. coli*, and *B. pumilus* with an MIC of 0.195 mg/mL. The ethanol and methanol extracts significantly reduced the percentage proliferation of DU-145, PC-3, and SKU-T cells at 100 μg/mL. These extracts demonstrated strong dose-dependent DPPH and NO scavenging and antibacterial and cell proliferation inhibition activities. The strong bioactivity of *P. glomerata* makes it a good candidate for the isolation and identification of active compounds for anticancer and related illnesses.

## 1. Background

Elephantiasis, commonly known as lymphatic filariasis (LF), is defined as a set of symptoms characterised by thickening of the skin and underlying tissues [1,2]. It is a painful and profoundly disfiguring disease that commonly affects the limbs, male genitalia, and female breasts. According to the scientific literature of medicine, the terms lymphatic filariasis and elephantiasis should not be used interchangeably; elephantiasis is a late manifestation of lymphatic filariasis [3]. Elephantiasis is characterised by swelling due to the accumulation of lymph fluid (lymphoedema). The two types of lymphoedema are primary lymphoedema, which is caused by a mutation in the genes involved in the development of lymphatics, and secondary lymphoedema, which is a result of filarial or non-filarial infection [4]. Filarial lymphoedema is caused by parasitic nematodes that occupy the lymphatic system, including the lymph nodes, whereas non-filarial lymphoedema is caused by complications from injury, repeated infection with streptococcus bacteria, sexually transmitted infections, tuberculosis, or cancers that affect the lymphatics [5]. The impairment of the lymph node function negatively affects the production of lymphocytes and macrophages, leading to a weakening of the immune system’s response to infection [6].

Cancer is a leading public health problem that continues to increase, but its burden is not uniform [7]. The burden continues to grow in both developed and developing countries due to a variety of factors, such as ageing and growth of the population, as well as increased prevalence of risk factors associated with economic transition, including smoking, obesity, physical inactivity, and reproductive behaviours [7,8]. Reports of cancer cases and mortality are rapidly growing each year [9]. Globally, cancers kill more people than acquired immunodeficiency syndrome (AIDS), tuberculosis, and malaria combined [10,11]. Estimates by the World Health Organisation (WHO) in 2015 reported cancer as the first or second leading cause of death before the age of 70 years in 91 of 172 countries, and it ranks third or fourth in the additional 22 countries [9]. Furthermore, the GLOBOCAN 2018 estimates of cancer incidence and mortality worldwide estimated 18.1 million new cancer cases (17.0 million excluding nonmelanoma skin cancer) and 9.6 million cancer deaths (9.5 million excluding nonmelanoma skin cancer) [9,12].

In Sub-Saharan Africa, cancer is a major public health problem, affecting many of its 1 billion inhabitants. It is reported to be among the three leading causes of premature death (at ages 30–69 years) in almost all constituent countries [9,13]. According to Morhason et al. [10], the Sub-Saharan African region is predicted to have a greater than 85% increase in cancer burden by 2030. Approaches to minimise the burden of cancer in Sub-Saharan Africa in the past few years have had little success because of low awareness of the cancer burden and a poor understanding of the potential for cancer prevention. Currently, there is no cure for cancer. Chemotherapy is the most used method for cancer management, especially in developed countries. However, the side effects of this procedure are severe and have also been reported to worsen cancer progression in most cases. Low to middle-income communities do not have access to such quality health facilities and cannot afford such treatments. Therefore, the majority still relies on medicinal plants for the management of various diseases, including elephantiasis, cancer, and associated conditions.

*Platycarpha glomerata* (Thunb.) Less. (also known as clustered Platycarpha, among other names) is one of the plant species that has recently become a species of interest. It is a perennial herbaceous plant from the Asteraceae family. It has a simple stem with a few branches, and its leaves are lanceolate, measuring 2–5 cm; it grows erect and can reach a height of 0.6 m. *P. glomerata* grows in the eastern portion of the country in inland areas of KwaZulu-Natal and the Eastern Cape, at elevations below 500 m, and flowers from November to early February [14]. It is commonly found in rural places, disturbed areas, and along dirt roads. In the wild, *P. glomerata* is known to thrive on poor, rocky soils in damp, humid places and disturbed areas. *P. glomerata* is cultivated as an ornamental plant and ground cover. It is also used to strengthen soil and prevent erosion [14]. Traditionally, the plant’s roots are also used as diuretics, for diarrhoea, itches (used topically), burns, boils, eye difficulties, joint discomfort, ulcers, and various skin disorders. The yellow sap is used to clean the ears [15], while the leaves are used for steaming painful legs, treating wounds and caner, and preventing visitors from fighting on premises during a ceremony or ritual [16].

*Platycarpha* is a relatively unknown genus of three species (*P. glomerata*, *P. carlinoides*, and *P. parvifolia*) endemic to southern Africa [17]. Of the three species, *P. glomerata* is widely used to treat cancer and elephantiasis by the Indigenous people and traditional healers of the Eastern Cape and KwaZulu-Natal Provinces of South Africa. However, scientific data regarding this highly utilised species in South Africa is limited. Therefore, the study was aimed at investigating the in vitro anticancer potential and phytochemical constituents of *P. glomerata*.

## 2. Results

### 2.1. Yield of Extraction

The percentage yields for *P. glomerata* root extract were hexane (0.98%), acetone (1.86%), ethanol (6.68%), methanol (3.40%) and water (9.28%).

### 2.2. Phytochemical Screening of Extracts

The qualitative phytochemical analysis of *P. glomerata* extracts revealed the presence of tannins, phlobatannins, saponins, flavonoids, steroids, terpenoids and cardiac glycosides, and the absence of alkaloids as shown in Table 1. (+)—positive; (−)—negative.

The spectroscopic methods of *P. glomerata* roots revealed the presence of five thiophenes and diol, while the areal part contained two germacrolides [18]. Triterpenes and flavonoids have also been detected [19].

### 2.3. Fourier-Transform Infrared Spectroscopy (FTIR)

**Figure 1:**
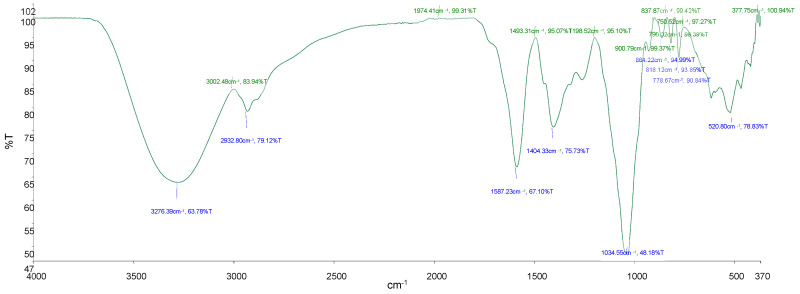
FTIR spectrum of aqueous extract from *P. glomerata*; %T: transmittance (%); cm^−1^: wavenumber (cm^−1^).

The FTIR analysis of *P. glomerata* aqueous extract showed the characteristic peaks at 3276.4 cm^−1^ H– bonded and O–H stretch, at 2932.8 cm^−1^ for asymmetric stretching of–CH(CH_2_) vibration, at 1587.2 cm^−1^ for C=C stretch, at 1404.3 cm^−1^ for O–H bend, alcoholic group, at 1034.6 cm^−1^ for PO_3_ stretch, and at 864.2 cm^−1^, 818.12 cm^−1^ and 778.7 cm^−1^ for =C–H bonding. Phytocompounds identified were hydroxy compounds, alcohols, and phenols. Saturated aliphatic compounds, lipids, aromatic compounds, phosphate ions and alkenes are shown in Figure 1 and Table 2.

### 2.4. In Vitro Antioxidant Assay

**Figure 2:**
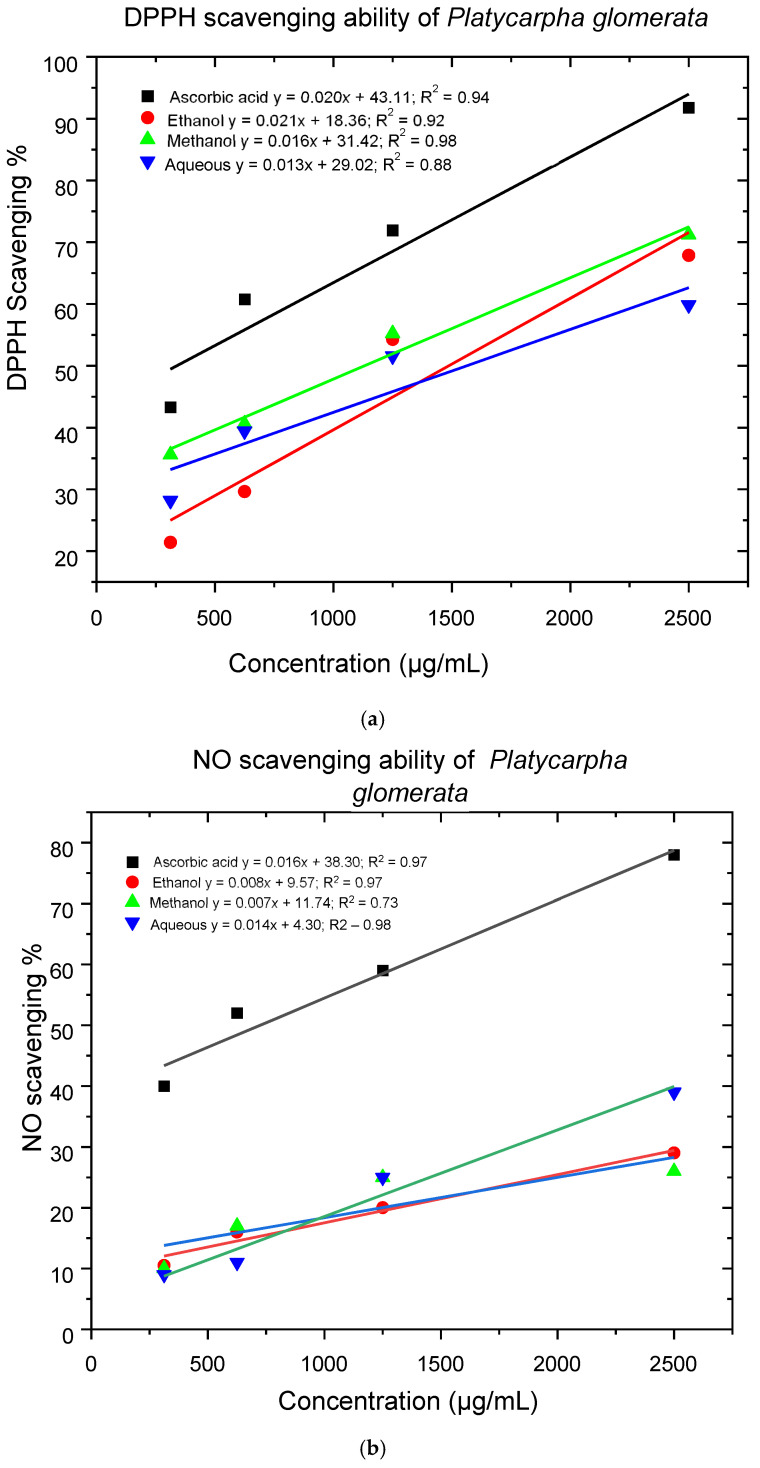
(**a**) DPPH radical scavenging activity of ethanol, methanol, and aqueous extracts. (**b**) NO scavenging activity of ethanol, methanol, and aqueous extracts.

The aqueous, ethanol, and methanol extracts of *P. glomerata* showed significant dose-dependent DPPH radical scavenging capacity (Figure 2a). The ascorbic acid was used as a standard control. The methanol extract was most efficient at 2500 µg/mL, inhibiting 31.4% of DPPH radical with the IC_50_ of 1161.3 ± 3.3 µg/mL, compared to ascorbic acid, which inhibited 43.1% at the same concentration, with an lC_50_ value of 344.5 ± 4.1 µg/mL (Table 3). Good antioxidant activity was also observed for the ethanol extract, with an 18.4% inhibition of DPHH radicals at 2500 µg/mL concentration and the lC_50_ value of 1506.67 ± 5.6 µg/mL. The aqueous extract demonstrated the least antioxidant activity, with 29.0% inhibition of DPHH radicals at 2500 µg/mL and an IC_50_ value of 1613.9 ± 3.9 µg/mL.

Figure 2b illustrates a minimal decrease in the NO radical due to the scavenging ability of aqueous, ethanol, methanol extracts and ascorbic acid. The aqueous, ethanol, and methanol extracts showed minimum activity of 4.3%, 9.6%, and 11.7% at 2500 µg/mL, with IC_50_ values of 3264.3 ± 6.5, 5053.8 ± 11.6, and 5465.7 ± 6.6 µg/mL, respectively, whereas ascorbic acid was 38.8% at the same concentration with the IC_50_ value of 731.3 ± 5.6 µg/mL (Table 3).

### 2.5. Antibacterial Activity

Table 4 shows the antibacterial activity results of *P. glomerata* methanol, ethanol, acetone, and aqueous extracts against *K. pneumoniae*, *S. aureus*, *E. coli*, and *B. pumilus*. Neomycin was used as a standard control. The water extract demonstrated good activity against *S. aureus*, *E. coli*, and *B. pumilus* at 0.195 mg/mL. Poor activity with the MIC value of 12.5 mg/mL was detected against *K. pneumoniae*. The ethanol extract displayed very good inhibition against the test bacterial strain between 0.098 and 0.39 mg/mL, except *B. pumilus*, which was inhibited at MIC of 1.56 mg/mL. The acetone and methanol extracts displayed potency against all test bacterial strains, with MIC values ranging between 0.098 and 0.78 mg/mL.

### 2.6. In Vitro Anticancer Activity

**Figure 3:**
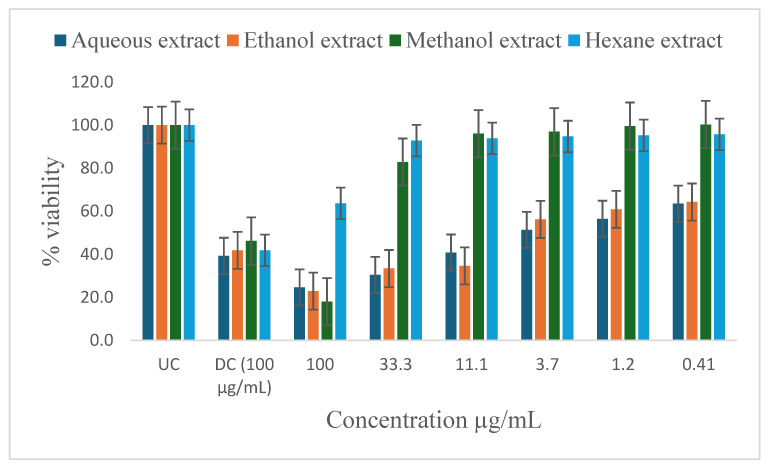
Inhibitory activity of *P. glomerata* extracts against DU-145 cell line measured as a percentage against untreated control and drug control. UC = untreated cell solution; DC = drug control; docetaxel (Taxotere). Error bars represent standard deviation calculated from three different experiments carried out in triplicate.

**Figure 4:**
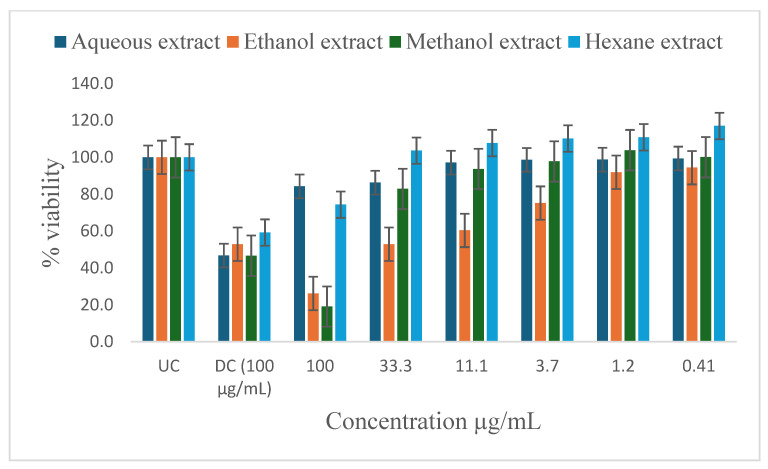
Inhibitory activity of *P. glomerata* extracts against PC-3 cell line measured as a percentage against untreated control and drug control. UC = untreated cell solution; DC = drug control; docetaxel (Taxotere). Error bars represent standard deviation calculated from three different experiments carried out in triplicate.

**Figure 5:**
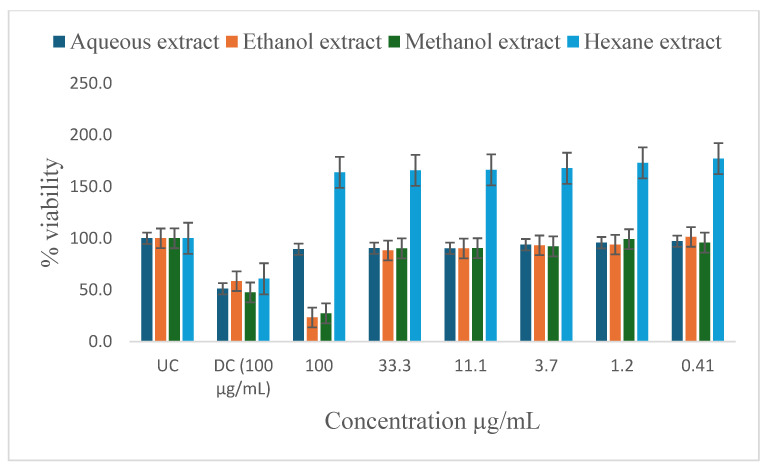
Inhibitory activity of *P. glomerata* extracts against SKU-T cell line measured as a percentage against untreated control and drug control. UC = untreated cell solution; DC = drug control; docetaxel (Taxotere). Error bars represent standard deviation calculated from three different experiments carried out in triplicate.

**Figure 6:**
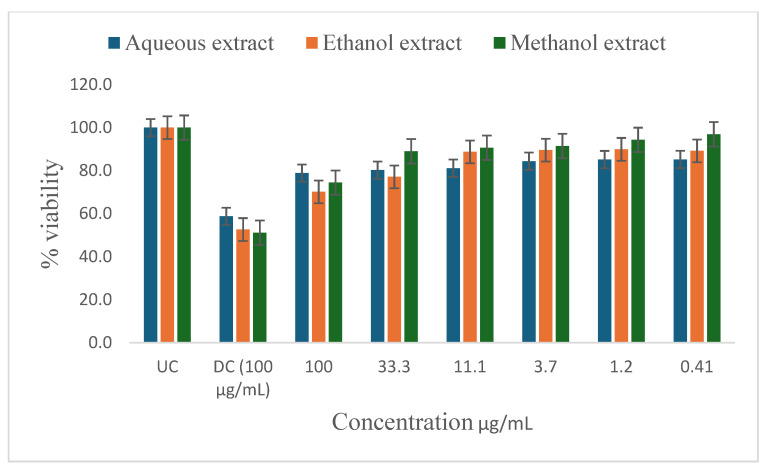
Inhibitory activity of *P. glomerata* extracts against AGS cell line measured as a percentage against untreated control and drug control. UC = untreated cell solution; DC = drug control; docetaxel (Taxotere). Error bars represent standard deviation calculated from three different experiments carried out in triplicate.

**Figure 7:**
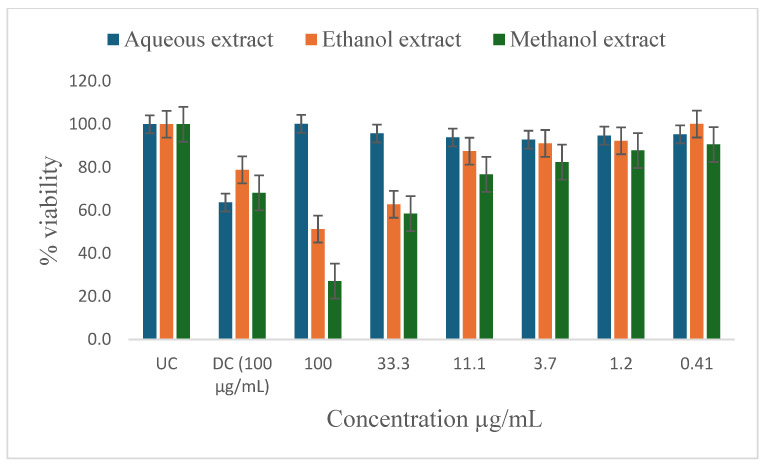
Inhibitory activity of *P. glomerata* extracts against PNTA1 cell line measured as a percentage against the untreated control and drug control. UC = untreated cell solution; DC = drug control; docetaxel (Taxotere). Error bars represent standard deviation calculated from three different experiments carried out in triplicate.

Table 5 shows the results of the IC_50_ and the percentage of inhibition of the plant extracts on cancer cells as determined by the MTT assay. In vitro antiproliferative effect of *P. glomerata* aqueous, ethanol, methanol, and hexane extract inhibition on prostate cancer cell lines (PC-3 and DU-145), human T-lymphocytes (SKU-T), gastric cancer cell lines (AGS), and human prostatic epithelial (PNTA1) cell lines are shown in Figure 3, Figure 4, Figure 5, Figure 6 and Figure 7. The untreated cell solution (UC) was used as a negative control, while the docetaxel (Taxotere) (DC) was used as a drug control.

The ethanol and methanol extracts significantly reduced the percentage viability of DU-145, PC-3, and SKU-T cells at 100 μg/mL (Figure 3, Figure 4 and Figure 5). The aqueous extract also significantly reduced the cell viability of the DU-145 cell line at 100 μg/mL (Figure 3). Significant reductions in DU-145 cell viability were observed in the lower concentrations of the ethanol extract, ranging from 0.41 to 11.1 μg/mL with the IC_50_ of 4.2 μg/mL and the percentage of inhibition of 91.8% (Table 5). In contrast, PC-3 cell lines showed a moderate response against the ethanol extract at 33.3 to 11.1 μg/mL and less to no response at 3.7 to 0.41 μg/mL concentrations with the IC_50_ of 2.3 μg/mL and the percentage of inhibition of 52.7%. The SKU-T cell line showed less to no inhibition to cell viability at 0.41 to 33.3 μg/mL. The hexane extracts significantly enhanced the viability of SKU-T cells at 100 to 0.41 μg/mL (Figure 5). The AGS showed less cell viability inhibition at 0.41 to 100 μg/mL (Figure 6). The PNTA1 cell line was also reduced at 33.3 to 100 μg/mL by ethanol and methanol extracts, whereas little to no response was observed at 0.41 to 3.7 μg/mL (Table 5 and Figure 7). DU-145, PC-3, and SKU-T showed no cell viability inhibition against the methanol extract from 0.41 to 33.3 μg/mL.

The hexane extracts moderately reduced the percentage viability of DU-145 and PC-3 cells at 100 μg/mL (Figure 3 and Figure 4). No activity was observed from 0.41 to 33.3 μg/mL. A rapid increase in cell proliferation of SKU-T on hexane extract was observed from 0.41 to 100 μg/mL (Figure 5). No reduction in the percentage viability of DU-145, PC-3, SKU=T, and AGS cell lines was observed in the aqueous extract from 0.41 to 100 μg/mL (Figure 6).

## 3. Discussion

As previously mentioned, there is limited information regarding bioassays, phytochemical assays, and medicinal uses of *P. glomerata* in the literature. The species is endemic to Southern Africa, and it is widely utilised by South African traditional healers and Indigenous people to treat various diseases, including elephantiasis, cancer, and related ailments [21]. This study is, however, the first to report on the phytochemical screening, FTIR analysis, and anticancer activity of *P. glomerata.*

### 3.1. Phytochemical Analysis

The phytochemical analysis of the *P. glomerata* extracts revealed the presence of phytochemicals such as tannins, flavonoids, saponins, steroids, terpenoids, and cardiac glycosides. The role of phytochemical constituents found in the same Asteraceae family that have been described and reviewed include tannins and phlobatannins [22], flavonoids [23], saponins [24], steroids [25], terpenoids [26], and cardiac glycosides [27]. Alkaloids were not detected in this study. The results are consistent with the findings reported by Nogueira da Silva et al. [28] in *Leishmania amazonensis*, a species of the Asteraceae.

### 3.2. Fourier Transform Infrared (FTIR) Spectroscopy Analysis

FTIR spectroscopy is a high-resolution analytical technique for identifying chemical constituents and analysing molecular structures. It provides a quick and non-destructive way to fingerprint plant extracts or powders [29]. As shown in (Table 2), the FTIR analysis of *P. glomerata* aqueous extract revealed the presence of the compounds and functional groups present in the species. C–F, O–H, C–H, C=C, C=O, C≡N, N–H, C–H, carbonate, and nitrate stretching were detected in *P. glomerata* (Table 2). The identified phytocompounds were hydroxy compounds, alcohols, phenols, saturated aliphatic compounds, lipids, aromatic compounds, phosphate ions, and alkenes (Figure 1 and Table 2). According to Paulraj et al. [30], all of these compounds belong to secondary plant metabolites. Similar findings on the plant species belonging to the Asteraceae family have been reported by various authors [31,32,33]. Fialová et al. [34] reported that phenolic compounds are an important group of active compounds in herbs since they act by disrupting the bacterium cell wall, interfering with the adenosine triphosphate (ATP) pool and altering its membrane potential, resulting in bacterium’s death. One aromatic chemical that has been researched for its possible anticancer benefits is resveratrol, which is present in red grapes and wine.

### 3.3. In Vitro Antioxidant Activity

The DPPH is a stable free radical molecule with delocalised electrons, giving its solution a purple colour upon interaction with antioxidant compounds or extracts containing antioxidants. DPPH is reduced to DPPH-H, turning the solution yellow as DPPH-H is formed. The extracts analysed in the DPPH assays demonstrated antioxidant activity. A lower or reduced IC_50_ value suggested better antioxidant activity. Ascorbic acid, a well-known antioxidant, had the lowest IC_50_ value among the samples tested. This indicated that ascorbate is the most effective at scavenging DPPH radicals, requiring only 344.50 ppm to achieve 50% inhibition. This result confirmed the strong antioxidant properties of ascorbic acid, making it a standard reference for comparing the antioxidant activity of other substances [35]. The methanol extract demonstrated good antioxidant activity, whereas the ethanol showed moderate antioxidant activity. Dewan et al. [36], Güneş et al. [37], and Piątkowska et al. [38] have reported similar findings of antioxidant activity on species in the Asteraceae family.

Nitric oxide is a potent pleiotropic inhibitor of physiological processes such as smooth muscle relaxation, neuronal signalling, inhibition of platelet aggregation, and regulation of cell-mediated toxicity [39]. The scavenging activity of NO by methanol, ethanol, and aqueous extracts was increased in a dose-dependent manner. The methanol and ethanol extracts showed minimal NO scavenging activity (Figure 2b). These findings are consistent with the study conducted by [39,40] on *Syzygium cumini* Linn. (Myrtaceae).

### 3.4. Antibacterial Activity

The antibacterial activity of specific concentrations of aqueous, ethanolic, methanolic, and acetone extracts of *P. glomerata* are presented in Table 3. The recorded MIC values demonstrated varying degrees of suppressing bacterial growth. All the extracts showed good activity against the tested bacteria, except for the ethanolic and water extracts against *B. pumilus* and *K. pneumoniae*, with MIC values of 1.56 mg/mL and 12.5 mg/mL, respectively. Penduka et al. [21] reported similar findings and concluded that *P. glomerata* could be a potential source of new antibacterial compounds/drugs for the treatment of skin and soft tissue bacterial infections. Extracts from other species of the Asteraceae family displayed good activity against some of the microorganisms in studies conducted by Nino et al. [41] and Kunte et al. [42]. In another study, members of the Asteraceae family showed small inhibition zones in a disc-diffusion assay [43].

### 3.5. Anticancer Activity

This study explored the in vitro antiproliferative effects of methanol, ethanol, and aqueous extracts of *P. glomerata* on prostate cancer cell lines (PC-3 and DU-145), human prostatic epithelial (PNTA1), human T-lymphocytes (SKU-T), and gastric cancer cell lines (AGS). The docetaxel (Taxotere) drug was used as a baseline. The evaluation revealed promising activities and concentration-dependent effects across different extracts. The ethanol and methanol extracts demonstrated significant antiproliferation effects against DU-145, PC-3, and SKU-T cell lines at higher concentrations with LC_50_ ranges from 0.8 to 4.2 μg/mL and the cell viability inhibition ranging from 62.1% to 91.8% at 100 μg/mL (Table 5). These results are consistent with the findings by Aouissi et al. [44], who reported on the anticancer activity of *Asteriscus graveolens* (Asteraceae) extracts against the HepG2 cell line; the findings reported 83% cell growth inhibition. The ethanol extract demonstrated antiproliferation against the DU-145 cell line, even at low concentrations. Comparing the antitumoral agents against human pancreatic cancer cells from Asteraceae and Lamiaceae plant extracts, Mouhid et al. [45] discovered that ethanol showed high levels of antiproliferation even at low concentrations compared to other extracts. The hexane extract demonstrated moderate antiproliferation activity against the DU-145 and PC-3 cell line at 100 μg/mL. However, the hexane extract enhanced the cell viability of SKU-T at all concentrations. This might be due to the lack of enough bioactive compound contents in the extract to trigger a response from the cells. The extract seems to have renewed or stimulated the media solution, promoting cell proliferation. The PNTA1 cell proliferation was only reduced by ethanol and methanol extracts at higher concentrations, whereas the AGS showed no responses against all the extracts except for the methanol extract at 100 μg/mL with moderate inhibition. The activity of the plant extracts may be attributed to the different classes of compounds found in the extracts [46]. A study undertaken by da Silva et al. [28] evaluated seven Brazilian plants of Asteraceae against cancer cells and *Leishmania amazonensis.* The results showed that among the 21 extracts tested, nine (43%) displayed moderate (50 to 75%) to high (N75%) in vitro antiproliferative activity against human cancer cells. Furthermore, the detected phytochemicals such as flavonoids, terpenoids, and steroids have been reported to possess antitumor or anticancer activities.

## 4. Materials and Methods

### 4.1. Collection of Plant Material

The *P. glomerata* plant species (whole plant) was collected from Durban and Komani in the KwaZulu-Natal and Eastern Cape Provinces of South Africa, respectively. Additional plant material was obtained from personnel working in nature conservation in Komani, Eastern Cape. Proper identification was completed by a taxonomist at the Faculty of Science and Agriculture, University of Fort Hare, South Africa.

### 4.2. Preparation of Extracts

The plants were kept at room temperature until dryness. About 30 g each of the dried powdered plant root material was extracted in 300 mL of ethanol, methanol, hexane, acetone, and distilled water by shaking on a Labcon platform shaker (Laboratory Consumables, PTY, Durban, South Africa) for 24 h. Extracts were filtered through Whatman No. 1 filter paper discs. The extraction was performed using five solvents of increasing polarity (hexane, acetone, ethanol, methanol, and aqueous). Hexane, acetone, ethanol, and methanol extracts were concentrated under reduced pressure at 45 °C using a rotary evaporator (Cole Parmer SB 1100, Shanghai, China), whereas the filtrate from the water extract was evaporated to dryness using a freeze–dryer (Genevac LTD, BTP-3ES00X, IP Swich, Ipswich, UK). All the crude extracts were stored at −20 °C until use.

Stock solutions of plant extracts for anticancer assays were prepared by dissolving 0.04 g crude extracts of ethanol, methanol, and hexane into a 2 mL mixture containing 0.1% dimethyl sulfoxide (DMSO), while the water crude extract was dissolved in 2 mL distilled water. All extracts were vortexed and passed through 0.45 μm and 0.22 μm sterile filters consecutively. The prepared aliquot extracts were wrapped with foil and stored at −20 °C until use.

### 4.3. Phytochemical Screening

The phytochemicals of *P. glomerata* were determined by adopting the standard methods as described by Harborne [47], Trease and Evans [48], Sofowora [49] and Edeoga [50]. The bark was tested for the presence of alkaloids, flavonoids, terpenoids, saponins, anthraquinones, cardiac glycosides, and tannins. The presence of phytochemicals was determined by visual observation of colour change or the production of a precipitate upon the addition of the prescribed reagent(s).

### 4.4. Fourier Transform Infrared Spectroscopy Analysis

The Fourier Transform Infrared (FTIR) Spectroscopic analysis was performed to identify the functional groups present in the plant’s extracts. FTIR was performed on the translucent sample disc. About 10 mg of the crude aqueous extract material was combined with 100 mg of KBr pellet and loaded into the FTIR spectroscope (Perkin Elmer Spectrum 100 FTIR spectrometer, Shelton, CT, USA). The FTIR spectroscope’s scan range was set from 400 to 4000 cm^−1^ with a resolution of 4 cm^−1^ for accurate analysis.

### 4.5. Antioxidant Assay

To measure the antioxidant activity of various plant extracts, each extract was screened using the 1-1-diphenyl-2-picrylhydrazyl (DPPH) radical scavenging and Nitric Oxide scavenging assays. The DPPH assay is a frequently used procedure for assessing the radical scavenging activity of antioxidants. It is claimed to be a reasonably stable free radical.

#### 4.5.1. DPPH Radical Scavenging Assay

The antioxidant properties of different plant solvent extracts were determined by DPPH scavenging activities. A volume of 2.5 mL of DPPH (2 mM) stock solution in methanol was gently mixed with five different concentrations (250 µg/mL, 125 µg/mL, 50 µg/mL, 10 µg/mL, 5 µg/mL) of each of plant extract, and the control (ascorbic acid) was prepared in methanol. A blank was also prepared. The solution was incubated for 30 min at room temperature before the absorbance was read with a spectrophotometer at 517 nm. Antioxidant properties were determined with Equation (1) below, as described by Madikizela and McGaw [51].

$$\%\;DPPH\;scavenging\;activity=\frac{Absorbance\;of\;sample-Absorbance\;of\;blank}{Absorbace\;of\;control-Absorbance\;of\;Blank}\times 100$$

where Absorbance of control is the absorbance of the DPPH radical + methanol; Absorbance of sample is the absorbance of DPPH radical + sample extract/standard.

#### 4.5.2. Nitric Oxide (NO) Scavenging Activity

The Nitric Oxide scavenging activity of the plant extracts was determined by the method outlined by Wintola and Afolayan [52]. A 2 mL sample of 10 mM sodium nitroprusside was prepared in phosphate-buffered saline (pH 7.4) and mixed with 0.5 mL of each extract together with standard solutions of BHT and gallic acid at different concentrations (50, 100, 200, 300, 400, 500 µg/mL). After the 2.5 h incubation of the samples at 25 °C, 0.1 mL of the incubated sample was combined with 0.1 mL of the Griess reagent [1.0 mL sulfanilic acid reagent (0.33%) prepared in 20% glacial acetic acid] and left at room temperature for 5 min. A 1 mL of naphthylenediamine dichloride (0.1% *w*/*v*) was added to the mixture and further incubated for 30 min at room temperature. The absorbance was read at 540 nm. The analysis was performed in triplicate. The amount of nitric oxide radicals inhibited by plant extracts was calculated using the following equation:

$$NO\;radical\;scavenging\;activity\left(\%\right)=\frac{Absorbance\;of\;sample-Absorbance\;of\;blank}{Absorbace\;of\;control-Absorbance\;of\;Blank}\times 100$$

where Absorbance of control is the absorbance of NO radicals + methanol and Absorbance of sample is the absorbance of NO radical + extract or standard.

### 4.6. Antibacterial Assay

*Escherichia coli* (ATCC 8739), *Staphylococcus aureus* (ATTC 6538), *Klebsiella pneumoniae* (ATTC 13047), and *Bacillus pumilus* (ATCC 14884) were obtained from [anonymised] and maintained on Mueller–Hinton (MH) agar. The bacterial agents were chosen as the most common wound and skin causal infections.

The microplate method of Eloff [53] was used to determine the minimal inhibitory concentration (MIC) values for plant extracts with antibacterial activity. Residues of plant extracts were dissolved at 50 mg/mL with the extracting solvents. All extracts were initially tested at 12.5 mg/mL in 96-well microplates and serially diluted two-fold to 0.098 mg/mL, after which 100 μL bacterial cultures were added to each well. Negative controls included the solvent used to dissolve the plant extracts and bacteria-free wells. All extracts were tested three times. The microplates were covered and incubated for 24 h at 37 °C. The MIC values were recorded as the lowest concentration of the extract that completely inhibited bacterial growth, i.e., a clear well. The microtiter plate reader was used to read the microplates at 600 nm, and the MIC values of each well were recorded.

### 4.7. Anticancer Activity

Human prostate carcinoma (DU-145 and PC-3), human T-lymphocyte (SKU-T), gastric adenocarcinoma (AGS) cancer cell lines, and human prostatic epithelial (PNTA1) cell lines obtained from the International Centre for Genetic Engineering and Biotechnology (ICGEB), Department of integrative Biomedical Sciences, South Africa were maintained in Dulbecco’s Modified Eagle Medium (DMEM) (ICGEB, Cape Town, South Africa) containing 10% fetal bovine serum (FBS), 1 mM L-glutamine, 100 units/mL penicillin, and 100 µg/mL streptomycin and kept at 37 °C in a humidified 5% CO_2_ incubator (Thermos Fisher Scientific, Frederick, MD, USA).

The visualisation of DU-145, PC-3, SKU-T, AGS, and PNTA1 cell viability was conducted according to a trypan blue staining method [54]. The concentration of cells per mL was determined using a haemocytometer, and the desired cell numbers were calculated using the following calculations:

Cells/mL = 10^4^ × (Average count per square) × (Dilution factor)

The plate was divided into the following three parts: complete media (blank), untreated cell solution as a control, and treatment with *P. glomerata* extract at different concentrations. The treatment was performed in triplicate.

The anticancer activity of the plant extracts was tested in vitro on DU-145, PC-3, SKU-T, AGS, and PNTA1 cell lines using a modified MTT (3-(4,5-dimethylthiazol-2-yl)-2,5-diphenyltetrazolium bromide) tetrazolium reduction assay as described [55]. Briefly, 6 × 10^4^ cells/well in 100 μL complete media were seeded into a 96-well cell culture microplate and allowed to attach to the plate for 24 h. After 24 h, the complete media was removed, and the cells were treated with the plant extract, of which 10 μg/mL was diluted to 200 μg/mL of complete media. Exactly 100 µg/mL of the diluted plant extract was added to the 96-well culture plate, and serial dilution was performed; untreated cell media solution was included as a control. The 96-well cell culture plates were then incubated at 37 °C in a humidified 5% CO_2_ for 72 h. Following incubation, 10 μL MTT (2.5 mg/mL) was added to each well and incubated for another 3–4 h and after sodium dodecyl sulfate 10% in 0.1 N HCl solution was added to solubilise the formed formazan and left overnight. The optical density in the wells was read in a microplate reader (Thermo Multiskan Go, Waltham, MA, USA) at a wavelength of 595 nm after 72 h [55]. The absorbance values obtained from the control wells were averaged, and this value was considered the 100% cell viability.

Cell viability was calculated as follows:

$$Percentage\;cell\;viability=\frac{Absorbance\;of\;sample}{Absorbance\;of\;control}\times 100\%$$

### 4.8. Statistical Analysis

Microsoft Excel 2013 was used for all statistical analyses. All experiments were performed in triplicate. An analysis of variance (ANOVA) was used to determine the statistical difference between the samples. The difference was considered statically significant at *p* < 0.05. The results were expressed as the mean ± standard deviation and compared using Duncan’s multiple range test.

## 5. Conclusions

*Platycarpha* is a relatively unknown genus of three species endemic to Southern Africa. *P. glomerata* is one of the species that has yet to be fully exploited for biological activities. In this study, extracts of *P. glomerata* displayed varying degrees of antiproliferation, antibacterial, and antioxidant activities. Notably, the plant showed promising activities against DU-145 and PC-3 prostate cancer cell lines. However, more research is necessary to assess the bioactivity of this species since factors such as the time of collection and the locality can also play a role in the biological activity of the plant.

## Figures and Tables

**Table 1:** Qualitative phytochemical screening of *P. glomerata*.

Plant Name	Extract Solvents	Tannins	Phlobatannins	Saponins	Flavonoids	Steroids	Terpenoids	Alkaloidal	Cardiac Glycosides
*P. glomerata*	Water	+	+	+	−	−	−	−	−
	Ethanol	+	−	−	+	+	−	−	+
	Methanol	+	−	−	−	−	−	−	−
	Acetone	−	−	+	+	−	+	−	+

(+)—positive; (−)—negative.

**Table 2:** Shows functional groups present in *P. glomerata* obtained from the FTIR analysis.

Spec. No.	Wave Number cm^−1^ (Test Samples)	Wave Number cm^−1^ [20]	Functional Group	Phyto Compounds Identified
**1**	3276.39	3570–3200	H– bonded, O–H stretch	Hydroxy compound, alcohols, phenols.
**2**	2932.80	2935–2915	Asymmetric stretching of—CH(CH_2_) vibration	Saturated aliphatic compound—Lipids.
**3**	1587.23	1600–1400	C=C stretch	Aromatic
**4**	1404.33	1410–1310	O–H bend, alcoholic group	Phenol or tertiary alcohol
**5**	1034.55	1100–1000	PO_3_ stretch	Phosphate ion
**6**	864.22	1000–675	=C–H bonding	Alkene
**7**	818.12	1000–674	=C–H bonding	Alkene
**8**	778.67	1000–674	=C–H bonding	Alkene
**9**	520.80	620–490	C-l	Halogen compound (Chloroform compound)

**Table 3:** Antioxidant activity of *P. glomerata* extracts (IC_50_ values in µg/mL). Ascorbic acid as a standard control.

Extracts	DPPH Assay (IC_50_ Values in µg/mL)	NO Assay (IC_50_ in µg/mL)
**Aqueous**	1613 ± 3 ^d^	3264 ± 6
**Ethanol**	1506 ± 5 ^c^	5053 ± 11
**Methanol**	1161 ± 3 ^b^	5465 ± 6
**Ascorbic acid**	344 ± 4 ^a^	731 ± 5

Data are expressed as mean ± standard deviation. Different letters (a–d) in the same column indicate significant differences among samples (*p* < 0.05), where *n* = 3.

**Table 4:** Antibacterial activity of *P. glomerata* extracts.

Plant Names	Extract Solvents	MIC Values of Different Bacterial Strains (mg/mL)			
		*K. pneumoniae*	*S. aureus*	*E. coli*	*B. pumilus*
*P. glomerata*	Methanol	0.098	0.098	0.098	0.78
	Ethanol	0.39	0.098	0.098	1.56
	Acetone	0.098	0.098	0.098	0.78
	Water	12.5	0.195	0.195	0.195
Neomycin (µg/mL)		0.098	0.098	0.098	0.098

**Table 5:** IC_50_ of the plant extracts on cancer cells as determined by the MTT assay.

Cell Lines										
	DU-145	PC-3	SKU-T	AGS	PNTA1	DU-145	PC-3	SKU-T	AGS	PNTA1
Plant Extracts	lC_50_ µg/mL					% Inhibition at 100 µg/mL				
Aqueous	4.2 ± 0.04	-	-	-	-	91.8	-	-	-	-
Ethanol	4.2 ± 0.14	2.3 ± 0.9	0.8 ± 0.05	-	0.3 ± 0.9	85.7	52.7	62.1	-	85.9
Methanol	1.1 ± 0.03	1.1 ± 0.6	0.4 ± 0.02	-	1.8 ± 0.9	93.4	91.3	87.4	-	92.6
Hexane	-	-	-	-	-		-	-	-	-

(-), Not found.

## Data Availability

All data generated or analysed during this study are included in this published article.
